# Supplementary material for: Saccharomyces boulardii (CNCM I-745) improves intestinal damage in sepsis by remodeling intestinal flora structure
Source: Front Cell Infect Microbiol. 2025 Jul 31;15:1602792. doi: 10.3389/fcimb.2025.1602792 (PMC12350329; doi:10.3389/fcimb.2025.1602792)
Supplement: Supplementary file 1 [file DataSheet1.zip › summary/summary/6_Taxonomy_community/1_Taxonomy_profiling/2_Phylum/Group/Phylum_abund_top30.Group.cluster.pdf]

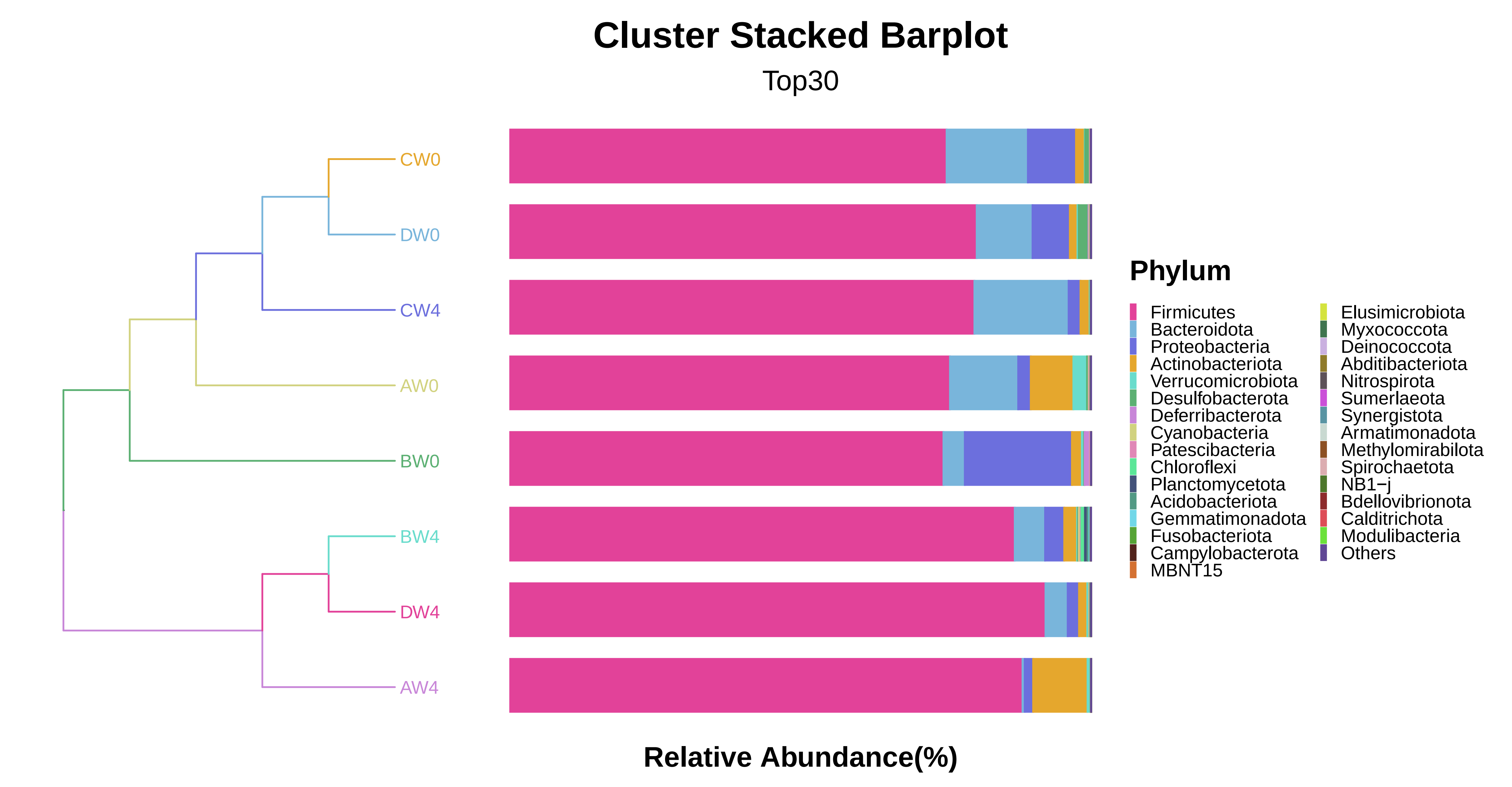

Cluster Stacked Barplot
Top30
CW0
DW0
CW4
AW0
BW0
BW4
DW4
AW4
Relative Abundance(%)
Phylum
Firmicutes
Bacteroidota
Proteobacteria
Actinobacteriota
Verrucomicrobiota
Desulfobacterota
Deferribacterota
Cyanobacteria
Patescibacteria
Chloroflexi
Planctomycetota
Acidobacteriota
Gemmatimonadota
Fusobacteriota
Campylobacterota
MBNT15
Elusimicrobiota
Myxococcota
Deinococcota
Abditibacteriota
Nitrospirota
Sumerlaeota
Synergistota
Armatimonadota
Methylomirabilota
Spirochaetota
NB1−j
Bdellovibrionota
Calditrichota
Modulibacteria
Others
